# Anidulafungin for the treatment of candidaemia/invasive candidiasis in selected critically ill patients

**DOI:** 10.1111/j.1469-0691.2012.03784.x

**Published:** 2012-01-30

**Authors:** M Ruhnke, J A Paiva, W Meersseman, J Pachl, I Grigoras, G Sganga, F Menichetti, P Montravers, G Auzinger, G Dimopoulos, M Borges Sá, P J Miller, T Marček, M Kantecki

**Affiliations:** 1 Department of Medicine, Charité University HospitalBerlin, Germany; 2 Department of Critical Care Medicine, Hospital de São JoãoPorto, Portugal; 3 Metabolic Diseases-Intensive Care Medicine, University Hospital LeuvenLeuven, Belgium; 4 Department of Anesthesiology and Critical Care Medicine, 3rd School of Medicine, Faculty Hospital Královské Vinohrady and Charles UniversityPrague, Czech Republic; 5 Department of Critical Care Medicine, University of Medicine and Pharmacy IaşiIaşi, Romania; 6 Department of Surgery, Catholic University Hosp A. GemelliRome, Italy; 7 Infectious Disease Unit, University Hospital PisaPisa, Italy; 8 Department of Anesthesiology and Surgical Critical Care, Hospital Bichat Claude BernardParis, France; 9 Institute of Liver Studies, King’s College HospitalLondon, UK; 10 2nd Department of Intensive Care Medicine, University Hospital ‘Attikon’Athens, Greece; 11 Intensive Care Unit, Hospital Son LlatzerPalma de Mallorca, Spain; 12 Specialty Care, Pfizer Global Research and DevelopmentSandwich, UK; 13 Specialty Care, Pfizer PIOParis, France

**Keywords:** *Candida*, Echinocandins, efficacy, global response, intensive care unit, safety

## Abstract

A prospective, multicentre, phase IIIb study with an exploratory, open-label design was conducted to evaluate efficacy and safety of anidulafungin for the treatment of candidaemia/invasive candidiasis (C/IC) in specific ICU patient populations. Adult ICU patients with confirmed C/IC meeting ≥1 of the following criteria were enrolled: post-abdominal surgery, solid tumour, renal/hepatic insufficiency, solid organ transplant, neutropaenia, and age ≥65 years. Patients received anidulafungin (200 mg on day 1, 100 mg/day thereafter) for 10–42 days, optionally followed by oral voriconazole/fluconazole. The primary efficacy endpoint was global (clinical and microbiological) response at the end of all therapy (EOT). Secondary endpoints included global response at the end of intravenous therapy (EOIVT) and at 2 and 6 weeks post-EOT, survival at day 90, and incidence of adverse events (AEs). The primary efficacy analysis was performed in the modified intent-to-treat (MITT) population, excluding unknown/missing responses. The safety and MITT populations consisted of 216 and 170 patients, respectively. The most common pathogens were *Candida albicans* (55.9%), *C. glabrata* (14.7%) and *C. parapsilosis* (10.0%). Global success was 69.5% (107/154; 95% CI, 61.6–76.6) at EOT, 70.7% (111/157) at EOIVT, 60.2% (77/128) at 2 weeks post-EOT, and 50.5% (55/109) at 6 weeks post-EOT. When unknown/missing responses were included as failures, the respective success rates were 62.9%, 65.3%, 45.3% and 32.4%. Survival at day 90 was 53.8%. Treatment-related AEs occurred in 33/216 (15.3%) patients, four (1.9%) of whom had serious AEs. Anidulafungin was effective, safe and well tolerated for the treatment of C/IC in selected groups of ICU patients.

## Introduction

Invasive *Candida* infections have a particularly strong impact on intensive care unit (ICU) patients [1], being associated with mortality rates of 30–50% [1,2]. Fluconazole is generally effective for candidaemia/invasive candidiasis (C/IC), but its use may be hampered by a potential increase in infections due to fluconazole-resistant *Candida* spp. [3–5]. Recent guidelines favour echinocandins as first-line therapy in haemodynamically unstable patients, those with previous azole exposure, and clinical settings with high local prevalence of fluconazole resistance [6–9]. However, the optimum therapy for C/IC in critically ill patients is unknown.

Anidulafungin has excellent activity against invasive isolates of *Candida* spp., including azole-resistant strains [10–12]. Anidulafungin was shown to be more effective than fluconazole for C/IC [13]; additional *post hoc* analyses seem to confirm its efficacy in critically ill patients [14]. However, prospective data on its use in this setting are lacking. Notably, less than half of all patients enrolled in previous clinical trials of echinocandins for C/IC were in the ICU at treatment initiation [14–16].

This exploratory, multicentre study prospectively evaluated efficacy and safety of intravenous (IV) anidulafungin, optionally followed by an oral azole, as first-line therapy for confirmed C/IC in selected ICU patient populations across Europe and Canada. The trial represents the first prospective assessment of an echinocandin for C/IC exclusively in ICU patients, who comprise a major target population for this antifungal class in clinical practice.

## Methods

### Study design

This was a phase IIIb, prospective, open-label, non-comparative study in adult (≥18 years) ICU patients from ≥1 of the following subpopulations: post-abdominal surgery, solid tumour, renal insufficiency, hepatic insufficiency, solid organ transplant, neutropaenia (neutrophil count < 500/mm^3^), and age ≥65 years. Eligible patients had an Acute Physiology and Chronic Health Evaluation II (APACHE II) score <25, signs and symptoms of acute invasive fungal infection (IFI) within 48 h before starting study treatment, and confirmed C/IC within 96 h before to 48 h after starting study treatment. Patients who had received antifungals for ≤48 h before study entry (one echinocandin dose maximum) without improvement were eligible. Presence of renal/hepatic insufficiency was determined by the investigator according to local guidelines. Patients with suspected *Candida* osteomyelitis, endocarditis, meningitis and/or endophthalmitis were excluded.

The study was conducted in accordance with the Declaration of Helsinki and Good Clinical Practice guidelines, and was approved by all appropriate institutional review boards/ethics committees. All patients or their legally authorized representatives were required to provide written informed consent.

### Treatment

Patients received IV anidulafungin (200 mg on day 1, then 100 mg/day) for 10–42 days. Patients completing ≥10 days’ treatment could be switched to oral voriconazole or fluconazole, provided they had two consecutive negative blood cultures and resolution of IFI signs and symptoms. Azole dosage was chosen according to local practice. Overall therapy (with anidulafungin or step-down azole) was continued for ≥14 days after the last positive blood/tissue culture and resolution/significant improvement of IFI signs and symptoms. The total maximum treatment duration was 56 days.

### Endpoints

The primary endpoint was global response at end of all therapy (EOT) in the modified intent-to-treat (MITT) population (i.e. patients with confirmed C/IC at study entry who received ≥1 anidulafungin dose). Global treatment success was defined as both clinical and microbiological success (i.e. cure/significant improvement of C/IC signs/symptoms and eradication/presumed eradication of *Candida* spp). Presumed eradication was defined as clinical success in the absence of microbiological cultures. Global response was defined as ‘missing’ or ‘unknown’ in all patients with missing or unknown clinical response, respectively, and any microbiological response except failure. Clinical response was defined as ‘unknown’ in unevaluable patients (i.e. death (not caused by C/IC), loss to follow-up, or received <3 anidulafungin doses). Unless stated, missing or unknown responses were excluded from analyses of global response. Secondary endpoints included global response at end of IV therapy (EOIVT) and at 2 and 6 weeks post-EOT, 90-day survival in the MITT population, and incidence of adverse events (AEs) in the safety population (i.e. patients who received ≥1 anidulafungin dose). *Candida* scores were determined at study entry [17]; calculation of the colonization index (i.e. number of positive sites/number of tested sites) was optional.

### Statistical analyses

This study was exploratory. Success rates are presented as number and percentage of patients with treatment success at each time-point, with exact two-sided 95% confidence intervals (CIs). Two-sided *Z*-tests were used to determine whether the proportions of treatment successes were significantly different between patients with and without baseline *C. albicans*, candidaemia or septic shock, by baseline APACHE II score (≤20 vs. >20) or treatment pathway (oral step-down therapy vs. anidulafungin alone), or by prompt intravascular catheter removal. Survival to day 90 and day of first negative blood culture were estimated using Kaplan-Meier methods.

## Results

### Patients and treatment

**FIG. 1:**
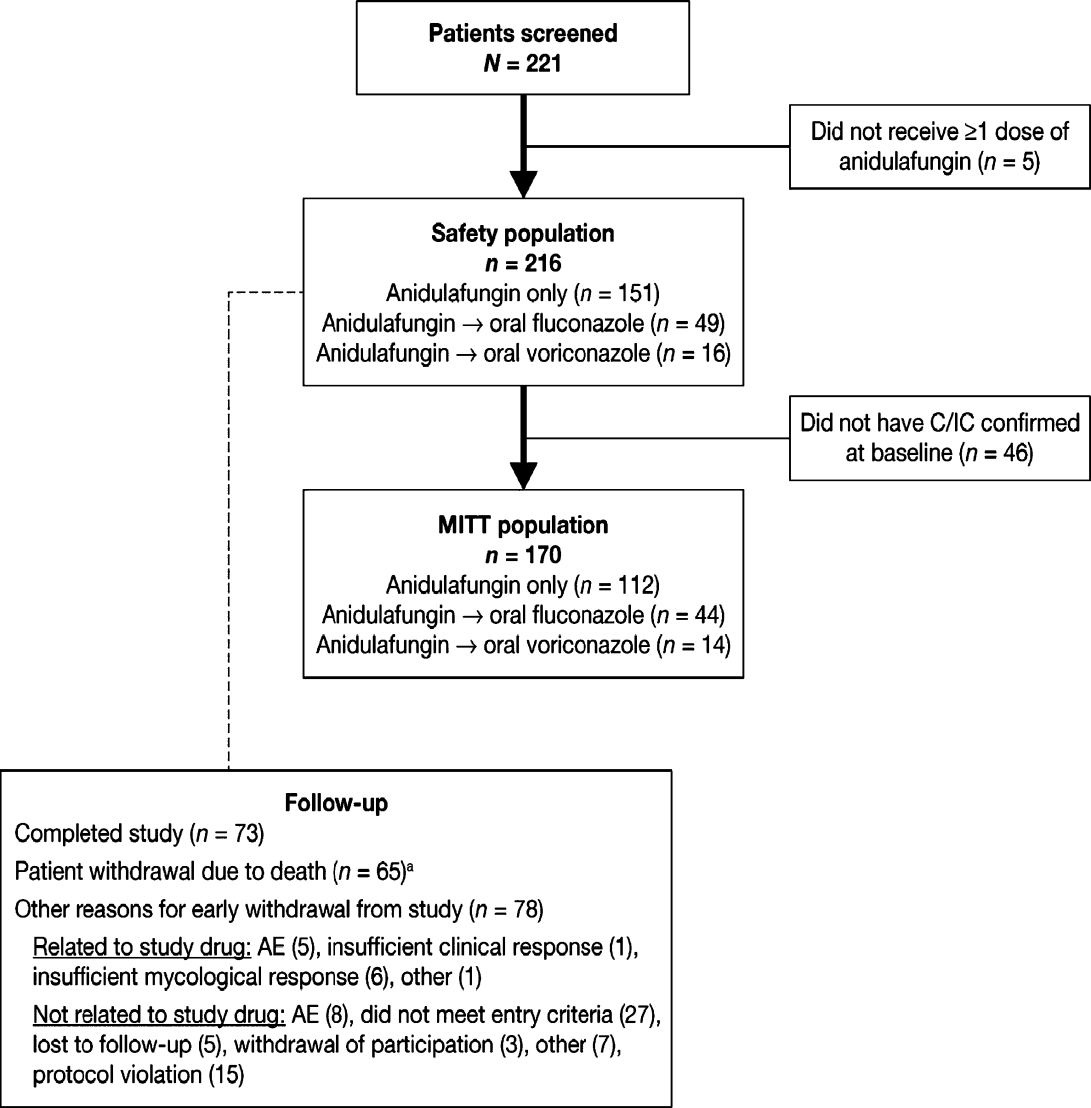
Patient flowchart. ^a^Until end of study, which could range from first day of study medication until 6 weeks after end of therapy depending on the specific patient.

A total of 221 patients were screened at 61 sites across 19 countries (Appendix S1). The safety and MITT populations comprised 216 and 170 patients, respectively (Fig. 1).

Baseline demographics and clinical characteristics of the MITT population are summarized in Table 1 and Appendix S1. Notably, 41 (24.1%) patients were in septic shock. Most patients had candidaemia only; the most common sites for deep-tissue infection were peritoneal fluid, bile and pleural fluid (Appendix S1). Most MITT patients fell into >1 ICU population at baseline: 34.1% patients were in two and 28.2% in three or four. All 216 safety population patients received concomitant drugs; commonly used co-medications were anticoagulants, anti-inflammatory agents, antimicrobials, benzodiazepines, diuretics, narcotic analgesics, proton-pump inhibitors and vasopressors.

**Table 1:** Baseline demographics and clinical characteristics of the modified intent-to-treat (MITT) population

Characteristic	MITT population (*n* = 170)
Demographic characteristics	
Male, *n* (%)	101 (59.4%)
Mean age (range)	62.2 years (25−89)
Race, *n* (%)	
White	160 (94.1%)
Other (includes unspecified)	10 (5.9%)
Mean BMI (range)^a^	25.7 kg/m^2^ (15.4–83.0)
Risk factors for candidaemia/invasive candidiasis, *n* (%)	
Broad-spectrum antibiotics	153 (90.0%)
Central venous catheter	148 (87.1%)
Prior surgery	113 (66.5%)
Total parenteral nutrition	99 (58.2%)
Dialysis/renal failure	59 (34.7%)
Systemic steroids or other immunosuppressives/immunosuppressive therapy	57 (33.5%)
Mucosal colonization by *Candida* species	52 (30.6%)
Chemotherapy	21 (12.4%)
Neutropaenia (neutrophil count <500/mm^3^)	13 (7.6%)
HIV infection	2 (1.2%)
Clinical characteristics	
Post-abdominal surgery	90 (52.9%)
Elderly (≥65 years)	80 (47.1%)
Renal insufficiency/failure/dialysis^b^	67 (39.4%)
Solid tumour	45 (26.5%)
Hepatic insufficiency^b^	27 (15.9%)
Neutropaenic	13 (7.6%)
Solid organ transplant recipient	10 (5.9%)
Infection site, *n* (%)	
Blood only	114 (67.1%)
Other normally sterile site only	49 (28.8%)
Blood and other normally sterile site	7 (4.1%)
Mean *Candida* score (95% CI)^c^	3.4 (3.2–3.6)
Mean colonization index (95% CI)^d^	53.1 (45.7–60.6)
Mean SOFA score (95% CI)^e^	7.2 (6.6–7.9)
Septic shock^f^	41 (24.1%)
APACHE II score	
≤20	128 (75.3%)
>20	42 (24.7%)
Mean (range)	16.2 (4–26^g^)
Intravascular catheter status	
All catheters removed/replaced^h^	40 (23.5%)
Not all catheters removed/replaced^i^	49 (28.8%)
No catheter inserted before first positive culture	81 (47.6%)
Baseline pathogen	
*C. albicans*	95 (55.9%)
*C. glabrata*	25 (14.7%)
*C. parapsilosis*	17 (10.0%)
*C. tropicalis*	13 (7.6%)
*C. kefyr*	3 (1.8%)
*C. dubliniensis*	2 (1.2%)
*C. pelliculosa*	2 (1.2%)
Other *Candida* spp.^j^	3 (1.8%)
Multiple *Candida* spp.	10 (5.9%)

APACHE II, Acute Physiology and Chronic Health Evaluation II; BMI, body mass index; SOFA, Sequential Organ Failure Assessment.

a Assessed in *n* = 165 patients.

b The presence/absence of these characteristics was determined by the local investigator; there were no prespecified protocol definitions.

c Assessed in *n* = 167 patients.

d Assessed in *n* = 90 patients, expressed as a percentage.

e Assessed in *n* = 166 patients.

f Defined as having ‘severe sepsis’ (per the *Candida* score assessment) and a value of 3 or 4 on the cardiovascular system component of the SOFA score.

g A single patient with a score ≥25 (i.e. 26) was included in the MITT population.

h Patients with ≥1 intravascular catheter inserted before the day of first positive culture, all of which were removed or replaced by day 3 of anidulafungin therapy.

i Patients with ≥1 intravascular catheters inserted before the day of first positive culture, ≥1 of which had not been removed or replaced by day 3 of anidulafungin therapy.

j One each of *C. krusei*, *C. lusitaniae* and *C. norvegensis.*

The predominant causative organism was *C. albicans*, followed by *C. glabrata* and *C. parapsilosis* (Table 1). A total of 167 baseline isolates underwent susceptibility testing, and most of these (*n* = 153) were fully susceptible to anidulafungin, fluconazole and voriconazole (Appendix S1). At treatment initiation, five MITT patients had a presumptive C/IC diagnosis (confirmed within 48 h), while the remainder had documented C/IC. In patients with candidaemia only, the mean time between first positive blood culture and start of anidulafungin therapy was 2.3 days.

The mean overall treatment duration in MITT patients was 19.9 days (median, 18.5; range, 1–67), with a mean duration of anidulafungin therapy of 15.9 days (median, 14; range, 1–42). A total of 112 MITT patients (65.9%) received anidulafungin only (mean duration, 16.2 days; range, 1–42), while 44 (25.9%) were switched to oral fluconazole (mean duration, 11.5 days; range, 1–44) and 14 (8.2%) to oral voriconazole (mean duration, 12.0 days; range, 4–30).

### Efficacy

**FIG. 2:**
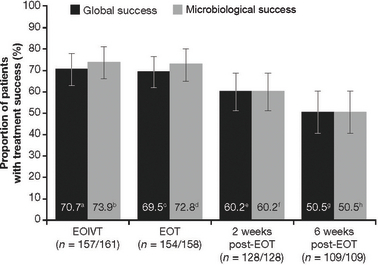
Global and microbiological success rates (with 95% confidence intervals) in modified intent-to-treat patients at the end of intravenous therapy (EOIVT), end of therapy (EOT), 2 weeks post EOT and 6 weeks post EOT. Missing and unknown global or microbiological responses were excluded in these analyses. ^a^95% confidence interval (CI), 62.9–77.7. ^b^95% CI, 66.4–80.5. ^c^95% CI, 61.6–76.6. ^d^95% CI, 65.1–79.6. ^e^95% CI, 51.1–68.7. ^f^95% CI, 51.1–68.7. ^g^95% CI, 40.7–60.2. ^h^95% CI, 40.7–60.2.

Global and microbiological success rates in the MITT population are shown in Fig. 2. Global success at EOT was 69.5% (107/154 patients; 95% CI, 61.6–76.6). If missing and unknown responses among MITT patients (*n* = 170) were treated as failures, global success rates decreased to 65.3% (95% CI, 57.6–72.4) at EOIVT, 62.9% (95% CI, 55.2–70.2) at EOT, 45.3% (95% CI, 37.7–53.1) at 2 weeks post-EOT, and 32.4% (95% CI, 25.4–39.9) at 6 weeks post-EOT.

No meaningful differences in global and microbiological success rates were evident (Table 2) amongst most ICU patient populations, with the possible exception of those with neutropaenia (*n* = 12) and solid organ transplants (*n* = 8), although the wide CIs in these populations limit the interpretation of these findings. Success rates were not significantly different in patients with and without candidaemia and were similar in patients with and without *C. albicans* (except for *C. tropicalis*). Global success rates throughout the study were also similar in patients with baseline APACHE II scores of ≤20 and >20 and in patients with or without septic shock (Table 3). Global success rates were significantly greater in patients receiving oral step-down therapy vs. those receiving anidulafungin alone (Table 3). For patients with successful global response at EOT, post-EOT success rates were similar when given anidulafungin only vs. anidulafungin followed by oral step-down therapy, at 2 (95.1% vs. 94.7%) and 6 weeks (89.7% vs. 87.1%). Global success at EOT in non-neutropaenic patients (*n* = 142) was 71.1% (95% CI, 62.9–78.4). In patients with intravascular catheters present before day of first positive culture, global success rate at EOT was higher for patients with all such catheters removed/replaced by day 3 of anidulafungin treatment (77.1%; 95% CI, 59.9–89.6) than otherwise (60.0%; 95% CI, 44.3–74.3), although the difference was not statistically significant (p 0.10). First negative blood culture was achieved by day 2 in >50% of evaluable patients (Appendix S1).

**Table 2:** Global and microbiological success in modified intent-to-treat patients at the end of therapy according to specific ICU patient population and baseline characteristics

	Global success, *n* (%) [95% CI]	Microbiologicl success, *n* (%) [95% CI]
ICU patient population		
Post-abdominal surgery	54/79 (68.4%) [56.9–78.4%]	55/80 (68.8%) [57.4–78.7%]
Elderly (≥65 years)	49/72 (68.1%) [56.0–78.6%]	54/75 (72.0%) [60.4–81.8%]
Renal insufficiency	44/58 (75.9%) [62.8–86.1%]	48/61 (78.7%) [66.3–88.1%]
Solid tumour	31/41 (75.6%) [59.7–87.6%]	32/42 (76.2%) [60.5–87.9%]
Hepatic insufficiency	18/25 (72.0%) [50.6–87.9%]	21/25 (84.0%) [63.9–95.5%]
Neutropaenic	6/12 (50.0%) [21.1–78.9%]	7/12 (58.3%) [27.7–84.8%]
Solid organ transplant recipient	3/8 (37.5%) [8.5–75.5%]	4/8 (50.0%) [15.7–84.3%]
Baseline pathogen^a^		
*C. albicans*^b^	64/86 (74.4%) [63.9–83.2%]	69/89 (77.5%) [67.4–85.7%]
*C. glabrata*	15/22 (68.2%) [45.1–86.1%]	15/22 (68.2%) [45.1–86.1%]
*C. parapsilosis*	10/15 (66.7%) [38.4–88.2%]	11/15 (73.3%) [44.9–92.2%]
*C. tropicalis*	4/11 (36.4%) [10.9–69.2%]	6/12 (50.0%) [21.1–78.9%]
Any non-*albicans*^b^	37/58 (63.8%) [50.1–76.0%]	40/59 (67.8%) [54.4–79.4%]
Baseline infection site		
Blood^c,d^	73/108 (67.6%) [57.9–76.3%]	81/112 (72.3%) [63.1–80.4%]
Other normally sterile site only^d^	34/46 (73.9%) [58.9–85.7%]	34/46 (73.9%) [58.9–85.7%]

Missing and unknown global or microbiological responses were excluded from these analyses.

a Excluding patients with multiple pathogens at baseline.

b The differences between success rates in patients with *C. albicans* and non-*albicans* infections were not statistically significant (p 0.17 for global response, p 0.19 for microbiological response).

c Includes patients with baseline infection site, either blood only or blood and other normally sterile site.

d The differences between success rates in patients with candidaemia and without candidaemia were not statistically significant (p 0.44 for global response, p 0.84 for microbiological response).

**Table 3:** Global success rates over the course of the study according to baseline APACHE II score, treatment strategy and septic shock status in modified intent-to-treat patients at the end of intravenous therapy (EOIVT), end of therapy (EOT), 2 weeks post-EOT and 6 weeks post-EOT

	EOIVT	EOT	2 weeks post-EOT	6 weeks post-EOT
APACHE II ≤20^a^				
*n* (%)	84/119 (70.6%)	80/116 (69.0%)	60/98 (61.2%)	44/84 (52.4%)
95% CI	61.5–78.6%	59.7–77.2%	50.8–70.9%	41.2–63.4%
APACHE II >20^a^				
*n* (%)	27/38 (71.1%)	27/38 (71.1%)	17/30 (56.7%)	11/25 (44.0%)
95% CI	54.1–84.6%	54.1–84.6%	37.4–74.5%	24.4–65.1%
Switched to oral azoles^b^				
*n* (%)	51/58 (87.9%)	47/55 (85.5%)	38/48 (79.2%)	29/41 (70.7%)
95% CI	76.7–95.0%	73.3–93.5%	65.0–89.5%	54.5–83.9%
IV anidulafungin only^b^				
*n* (%)	60/99 (60.6%)	60/99 (60.6%)	39/80 (48.8%)	26/68 (38.2%)
95% CI	50.3–70.3%	50.3–70.3%	37.4–60.2%	26.7–50.8%
Septic shock^a^				
*n* (%)	27/36 (75.0%)	25/34 (73.5%)	14/25 (56.0%)	10/22 (45.5%)
95% CI	57.8–87.9%	55.6–87.1%	34.9–75.6%	24.4–67.8%
No septic shock^a^				
*n* (%)	84/121 (69.4%)	82/120 (68.3%)	63/103 (61.2%)	45/87 (51.7%)
95% CI	60.4–77.5%	59.2–76.5%	51.1–70.6%	40.8–62.6%

Missing and unknown global or microbiological responses were excluded from these analyses.

a Differences between global success rates were not statistically significant (p > 0.05) at any time-point.

b Differences between global success rates were statistically significant (p < 0.05) at all time-points.

### Safety and survival

Among the 216 patients in the safety population, 151 (69.9%) received anidulafungin only; 49 (22.7%) and 16 (7.4%) also received step-down therapy with fluconazole or voriconazole, respectively. Treatment-related AEs occurred in 33/216 (15.3%) patients (total 80 events); most frequent were erythema (*n =* 4, 1.9%), hypotension, increased blood alkaline phosphatase, increased aspartate aminotransferase, diarrhoea and atrial fibrillation (each *n =* 3, 1.4%). Most treatment-related AEs were mild to moderate in severity (Appendix S1). Furthermore, only 1.9% of patients experienced serious treatment-related AEs (convulsions, *n =* 2; infusion-related AE, *n =* 1; bronchospasm, *n =* 1). The types and frequency of AEs were similar in the overall safety population and in patients who received anidulafungin only (Appendix S1). Six patients experienced ≥1 AE considered to potentially be infusion related. Overall, five (2.3%) patients were permanently discontinued from the study due to ≥1 treatment-related AE.

The 60-day and 90-day survival estimates in the MITT population were 58.0% (95% CI, 50.2–65.0) and 53.8% (95% CI, 45.9–60.9; Appendix S1), respectively.

## Discussion

This was the first prospective evaluation of therapy for C/IC conducted specifically in ICU patients. This exploratory non-comparative clinical trial confirmed the efficacy and safety of anidulafungin for the treatment of documented C/IC in selected adult ICU populations, many patients suffering from multiple co-morbidities. The global success rate at EOT was high (69.5%) and outcomes at this time-point were mostly similar regardless of ICU population, causative pathogen, infection site or clinical factors (including APACHE II score and septic shock status). Microbiological success rates were similar to the respective global successes. The overall incidence of treatment-related AEs (most were mild to moderate) was low, suggesting excellent tolerability of anidulafungin even in critically ill patients.

Published *post hoc* analyses of randomized clinical trials showed lower or similar success rates at EOT with fluconazole (54%), conventional amphotericin B (69%), liposomal amphotericin B (66%), micafungin (63%) and caspofungin (68%) in ICU patients with C/IC [14–16]. A similar *post hoc* analysis showed a 69% global success rate with anidulafungin in ICU patients at EOIVT, compared with 76% in the overall population of that study; both analyses treated missing and unknown responses as therapeutic failures [13,14] while our study excluded them from the primary endpoint. When these cases were counted as failures, the EOIVT global success rate in our study (65%) was almost identical to that reported in the ICU *post hoc* analysis with anidulafungin [14]. Of note, mean APACHE II scores were somewhat higher in the present study (16.2) than in the general C/IC population (15.0) [13]. Patients with APACHE II scores ≥25 were excluded from our trial, because the high crude mortality rate in such patients would have impacted the evaluation of drug efficacy; in patients with APACHE II scores >25 [18] or even >20 [13,19], treatment differences are no longer detectable. Exclusion of patients with high baseline scores is likely to have contributed to similar responses regardless of APACHE II score, contrary to what was observed in some previous studies [18–20].

Treatment duration was longer than in prospective trials assessing echinocandins for C/IC in general patient populations [13,19–21]; ICU patients may require longer durations of antifungal treatment than non-ICU patients [22]. Our results suggest that survival and global response rates in critically ill patients were lower at all time-points than previously observed in a general population [13]. This is consistent with other analyses indicating that ICU patients with invasive *Candida* infections have higher mortality and worse outcomes regardless of the antifungal agent used [14–16], probably reflecting the worse underlying condition of this population.

ICU patients with systemic *Candida* infections should therefore receive the most effective antifungal therapy available, as early as possible. Because the rapidly fungicidal action of echinocandins may positively impact treatment outcomes [23], these agents are now generally recommended as first-line therapy for C/IC in moderately to severely ill patients [8,9]. The results of our study support these clinical guidelines. Anidulafungin is the only echinocandin without dose adjustment requirements for renal and hepatic impairment, and with no known drug–drug interactions [24,25]. In our trial, patients with hepatic and/or renal insufficiency (including patients on dialysis) responded just as well as ICU patients overall, further supporting the potential value of anidulafungin in patients with organ dysfunction. Even though all patients received concomitant medications, the tolerability of anidulafungin was excellent.

Our study included a significant proportion (about one-third) of C/IC patients with deep-tissue infection. This proportion is considerably larger than in previous echinocandin trials in C/IC, including *post hoc* analyses in ICU patients [13–16,19–21], and supports the efficacy of anidulafungin for treating invasive candidiasis as well as candidaemia. Also noteworthy is that anidulafungin was just as effective against *C. parapsilosis* as against other species. This particular pathogen has somewhat higher echinocandin minimum inhibitory concentrations (MICs) than other *Candida* species, although the clinical significance of these findings is unknown [9,26,27]. The treatment response for *C. tropicalis* was lower than for other *Candida* species and also lower than reported previously with anidulafungin for *C. tropicalis* [13]. However, our sample size for this subpopulation was small. The observed species distribution matched what would be expected from a pan-European study [28–31] and MICs were similar to those reported previously [13].

Our results support the potential utility of the *Candida* score for early diagnosis of C/IC in ICU patients, with scores generally higher than the previously defined threshold of 2.5 [17]. This study also assessed the efficacy of de-escalation therapy in critically ill populations (i.e. switching from an echinocandin to oral azoles after resolution of clinical and microbiological signs of infection). The higher global success rates among patients receiving step-down therapy compared with those receiving anidulafungin alone was expected, because the study protocol dictated that only patients who responded to IV therapy could switch to oral azoles. Notably, among patients with treatment success at EOT, response rates were similar at later time-points regardless of the treatment strategy, suggesting similar efficacy of both approaches. Intravascular catheter removal by day 3 of therapy did not significantly impact treatment response; because indwelling catheters were not assessed for being a potential source of infection, this particular result should be treated with caution.

Our trial has several limitations. Due to the study design, no direct comparison of anidulafungin with another antifungal treatment is available. Furthermore, some of the specific subgroups comprised only a few patients, for example those with solid organ transplants, neutropaenia and *C. tropicalis* infections. The small sample sizes do not allow meaningful conclusions to be drawn about these specific populations.

In conclusion, this is the first clinical trial to prospectively evaluate an echinocandin in specific ICU populations, albeit using an exploratory approach. The results demonstrate that anidulafungin is an effective and safe treatment for confirmed C/IC (including deep-tissue infection) in critically ill patients, with success rates similar to those achieved with anidulafungin in a general population. This efficacy appears to remain consistent across certain high-risk patient groups, regardless of a multitude of clinical factors and the causative pathogen. Our observations support current guidelines [6–9] recommending echinocandins as first-line therapy for the treatment of C/IC in moderately to severely ill patients.

## Transparency Declaration

This study was sponsored by Pfizer. Editorial support was provided by Dominik Wolf at PAREXEL and was funded by Pfizer International Operations. M. Ruhnke has received research funding from MSD, Pfizer and Stifterverband für die Deutsche Wissenschaft; has received funds for speaking from Astellas, Essex, Gilead, MSD and Pfizer; and has received funds for advisory board membership from Astellas, Essex, Gilead, MSD and Pfizer. .-A. Paiva has received research funding from MSD, Novartis and Pfizer; has received funds for speaking from MSD, Novartis and Pfizer; and has received funds for advisory board membership from MSD, Novartis and Pfizer. W. Meersseman has received research funding from MSD and Pfizer; has received funds for speaking from MSD; and has received funds for advisory board membership from Pfizer. I. Grigoras has received funds for speaking from Abbott, Astra-Zeneca, Bayer, Eli Lilly, Fresenius, Merck and Pfizer; and has received funds for advisory board membership from Pfizer. J Pachl: nothing to declare. G. Sganga has received funds for speaking from Pfizer; and has received funds for advisory board membership from Pfizer. F. Menichetti: nothing to declare. P. Montravers has received funds for speaking from Astellas, MSD and Pfizer; and has received funds for advisory board membership from Astellas, MSD and Pfizer. G. Auzinger has received funds for speaking from Pfizer; and has received funds for advisory board membership from Pfizer. G. Dimopoulos has received research funding from Pfizer; has received funds for speaking from Pfizer; and has received funds for advisory board membership from Pfizer. M. Borges Sá has received funds for speaking from Astellas, Astra-Zeneca and Pfizer. P. J. Miller is an employee of Pfizer. T. Marček is an employee of Pfizer. M. Kantecki is an employee of Pfizer.

## Supporting Information

**Appendix S1.** Online supplementary appendix (contains full investigator list and detailed results on: susceptibility testing, prior antifungal treatment, specific sites of deep-tissue infection, time to negative blood culture, and treatment-related adverse events).
