# Supplementary material for: Anidulafungin for the treatment of candidaemia/invasive candidiasis in selected critically ill patients
Source: Clin Microbiol Infect. 2012 Jan 30;18(7):680–7. doi: 10.1111/j.1469-0691.2012.03784.x (PMC3510306; doi:10.1111/j.1469-0691.2012.03784.x)

# ONLINE SUPPLEMENTARY APPENDIX

Article title: Anidulafungin for the treatment of candidaemia/invasive candidiasis in selected critically ill patients

Journal: *Clinical Microbiology and Infection*

Authors: Markus Ruhnke, José-Artur Paiva, Wouter Meersseman, Jan Pachl, Ioana Grigoras, Gabriele Sganga, Francesco Menichetti, Philippe Montravers, Georg Auzinger, George Dimopoulos, Marcio Borges Sá, Paul J. Miller, Tomáš Marček, Michal Kantecki

Corresponding author:
Prof. Markus Ruhnke, MD
Department of Medicine, Charité Campus Mitte Berlin, Charitéplatz 1, 10117 Berlin, Germany
Tel: +49 30 450 513102; Fax: +49 30 450 513907
e-mail: markus.ruhnke@charite.de

**The following investigators participated in the ICE study:**

Halis Akalin (Turkey), Bernard Allaouchiche (France), Massimo Antonelli (Italy), Georg Auzinger (United Kingdom), Aplay Azap (Turkey), George Baltopoulos (Greece), Natalia Beloborodova (Russian Federation), Nicolas Bruder (France), Pierre Bulpa (Belgium), Elisabetta Cerutti (Italy), Volodymyr Cherniy (Ukraine), Paula Couthino (Portugal), Franceso Cristini (Italy), Pierre Damas (Belgium), Georgios Dimopoulos (Greece), Natalia Dmitrieva (Russian Federation), Lubos Drgona (Slovakia), Herve Dupont (France), Filomena Faria (Portugal), Jozef Firment (Slovakia), Wojciech Gaszynski (Poland), Ioana Grigoras (Romania), Shariq Haider (Canada), Gilles Hilbert (France), Zsolt Ivanyi (Hungary), Jan Jahoda (Czech Republic), Tom Jensen (Denmark), Boris Jung (France), Nikolay Klimko (Russian Federation), Galina Klyasova (Russian Federation), Iftihar Koksal (Turkey), Christine Lee (Canada), Abhiram Mallick (United Kingdom), Jean Mantz (France), Wouter Meersseman (Belgium), Francesco Menichetti (Italy), Martin Mistrik (Slovakia), Eduardo Monteiro (Portugal), Philippe Montravers (France), Ludmi Novitskaja-Usenko (Ukraine), José Artur Paiva (Portugal), Jan Pachl (Czech Republic), Rene Pelletier (Canada), Elisabeth Presterl (Austria), Michiel van Rijn (The Netherlands), Markus Ruhnke (Germany), Faouzi Saliba (France), Jens Schierbeck (Denmark), Gabriele Sganga (Italy), Herbert Spapen (Belgium), Dana Tomescu (Romania), Stefan Utzolino (Germany), Omrum Uzun (Turkey), Jean-Luis Vincent (Belgium), Barbara Volgyes (Hungary), Vincenzo Vullo (Italy), Jan de Waele (Belgium), Richard Wenstone (United Kingdom), Gabriele Wöbker (Germany), Arthur van Zanten (The Netherlands), Vaclav Zvonicek (Czech Republic).

## Participating countries and number of treated patients

Austria (2 patients treated)

Belgium (24 patients treated)

Canada (9 patients treated)

Czech Republic (13 patients treated)

Denmark (2 patients treated)

France (33 patients treated)

Germany (22 patients treated)

Greece (3 patients treated)

Hungary (8 patients treated)

Italy (29 patients treated)

The Netherlands (8 patients treated)

Poland (4 patients treated)

Portugal (12 patients treated)

Romania (10 patients treated)

Russian Federation (12 patients treated)

Slovakia (4 patients treated)

Turkey (11 patients treated)

Ukraine (3 patients treated)

United Kingdom (7 patients treated)

## Susceptibility testing

### Methods

Antifungal susceptibility testing for baseline isolates was conducted according to standard Clinical and Laboratory Standards Institute (CLSI) methods and breakpoints (i.e. anidulafungin non-susceptibility >2 µg/mL; fluconazole resistance ≥64 µg/mL; voriconazole resistance ≥4 µg/mL) [1,2].

### Results

A total of 167 baseline isolates underwent susceptibility testing, and most of these (*n* = 153) were fully susceptible to anidulafungin, fluconazole, and voriconazole. However, 2/96 *Candida albicans* isolates were not susceptible to both fluconazole and voriconazole, 4/27 *C. glabrata* were not susceptible to fluconazole, 5/21 *C. parapsilosis* were not susceptible to fluconazole and 1/21 were not susceptible to anidulafungin, and 2/2 *C. krusei* were not susceptible to fluconazole.

Overall minimum inhibitory concentrations required to inhibit the growth of 50% of organisms ($MIC_{50}$) and of 90% of organisms ($MIC_{90}$) for anidulafungin, fluconazole, and voriconazole were 0.03 and 0.5, 0.5 and 8, and ≤0.015 and 0.5 µg/mL, respectively. The anidulafungin $MIC_{90}$ for *C. parapsilosis* was 2 µg/mL higher than for other species (i.e. 0.03–0.12 µg/mL).

### References

1. Clinical and Laboratory Standards Institute (CLSI) (2008) Reference Method for Broth Dilution Antifungal Susceptibility Testing of Yeasts; Approved Standard - Third Edition. Wayne, PA: Clinical and Laboratory Standards Institute. CLSI document M27-A3 (ISBN 1-56238-666-2).

2. Clinical and Laboratory Standards Institute (CLSI) (2008) Reference Method for Broth Dilution Antifungal Susceptibility Testing of Yeasts; Third Informational Supplement. Wayne, PA: Clinical and Laboratory Standards Institute. CLSI document M27-S3 (ISBN 1-56238-667-0).

**Prior systemic antifungals at baseline in the modified intent-to-treat (MITT) population**

| | |
|---|---|
| **Prior systemic antifungal treatment, n (%)** | **69 (40.6)** |
| Fluconazole | 54 (31.8) |
| Caspofungin | 10 (5.9) |
| Voriconazole | 6 (3.5) |
| Amphotericin B | 2 (1.2) |

**Sites of deep-tissue Candida infection at baseline in the MITT population**

| Site | No. of patients (*n* = 170) |
|---|---|
| **Sterile site (other than blood) only** | **49** |
| Peritoneal fluid | 29 |
| Bile | 4 |
| Pleural fluid | 3 |
| Kidney | 1 |
| Other | (1 each) |
| Abdominal liquid puncture | |
| Aorta biopsy | |
| Intra-abdominal abscess | |
| Pancreas fluid | |
| Pre-vertebral abscess swab | |
| Pulmonary abscess | |
| Rectus fascia | |
| Tissue, lower shaft of hip | |
| Wound fluid, from bile duct | |
| >1 site of baseline *Candida* infection | 3 |
| **Blood and other sterile site** | **7** |
| Peritoneal fluid | 4 |
| Other | (1 each) |
| Abdominal fluid | |
| Ascites | |
| Pancreas | |

## Time to negative blood culture

The time (in days) from start of study drug to first negative blood culture (until the end of anidulafungin treatment) was assessed in those patients who had *Candida* bloodstream infection only (not including those patients who also had a deep-tissue *Candida* infection). Patients who received less than 3 days of anidulafungin were excluded. As a result of this narrow definition, only 98 patients were eligible for inclusion into this analysis. Moreover, any negative blood cultures on the first day of dosing were ignored in order to prevent the possibility of the first negative culture being recorded as occurring on the same day as initiation of study drug. By definition, it was thus assumed that a patient was still infected with *Candida* on the first day of dosing and that no patient had eradication on Day 1 of treatment. Out of this group, 87 patients had a first negative blood culture not followed by a positive blood culture in the next 3 days (or 4 days if this negative was observed on or after Day 10) whilst on anidulafungin treatment. Mean (SD) day of first negative blood culture in these 87 patients was 3.7 (3.07), range 2-17. A Kaplan-Meier curve (see Figure S1) shows that >50% of the 98 eligible patients achieved a first negative blood culture by Day 2.

**Figure S1. Kaplan-Meier estimate of day of first negative blood culture in eligible modified intent-to-treat patients**

Day 1 represents the first day of anidulafungin therapy.

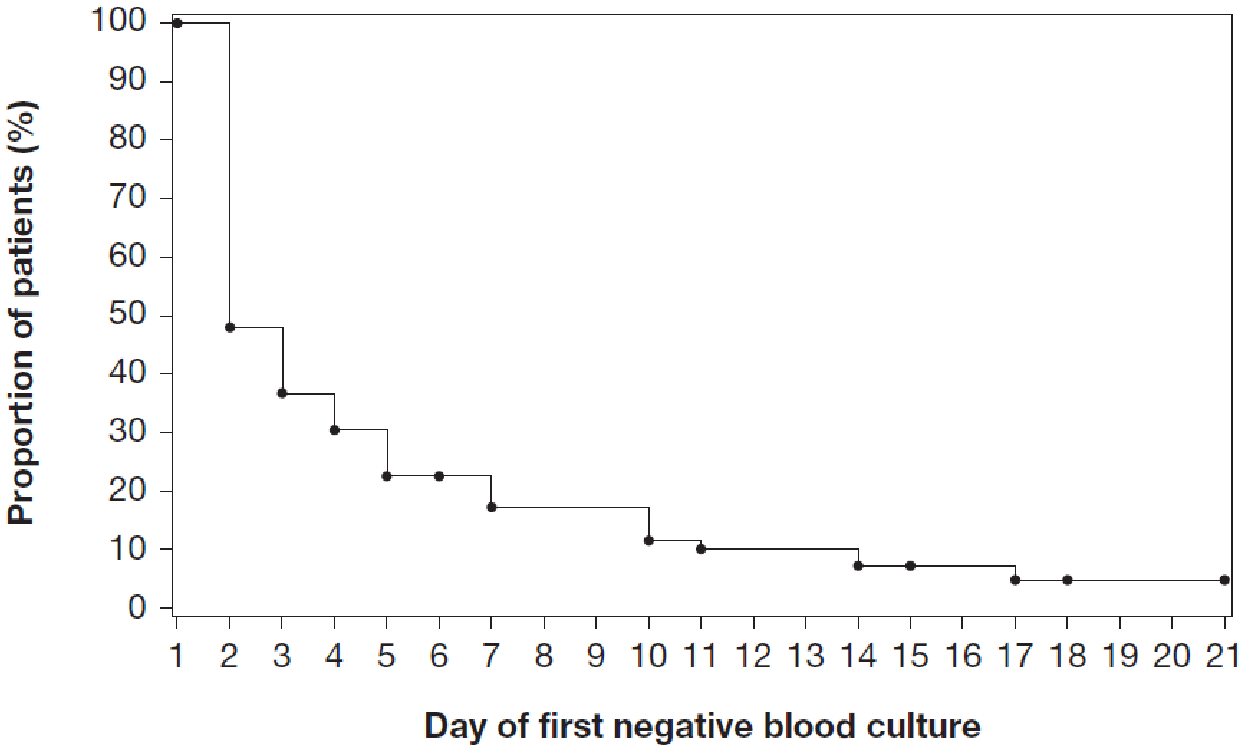

**Incidence of treatment-related adverse events in the overall safety population (n = 216)**

| Adverse event | *n* | (%) | Severity | | |
|---|---|---|---|---|---|
| | | | Mild | Moderate | Severe |
| **Blood and lymphatic system disorders** | 4 | 1.9 | 1 | 2 | 1 |
| Anaemia | 1 | 0.5 | 1 | 0 | 0 |
| Eosinophilia | 1 | 0.5 | 1 | 0 | 0 |
| Thrombocytopaenia | 2 | 0.9 | 0 | 2 | 0 |
| Thrombocytosis | 1 | 0.5 | 0 | 0 | 1 |
| **Cardiac disorders** | 7 | 3.2 | 5 | 1 | 1 |
| Atrial fibrillation | 3 | 1.4 | 2 | 1 | 0 |
| Bradycardia | 1 | 0.5 | 1 | 0 | 0 |
| Cardiac failure congestive | 1 | 0.5 | 1 | 0 | 0 |
| Myocardial ischaemia | 1 | 0.5 | 1 | 0 | 0 |
| Supraventricular tachycardia | 1 | 0.5 | 1 | 0 | 0 |
| Tachycardia | 2 | 0.9 | 1 | 0 | 1 |
| **Gastrointestinal disorders** | 6 | 2.8 | 4 | 2 | 0 |
| Abdominal pain | 2 | 0.9 | 2 | 0 | 0 |
| Diarrhoea | 3 | 1.4 | 2 | 1 | 0 |
| Nausea | 1 | 0.5 | 1 | 0 | 0 |
| Vomiting | 2 | 0.9 | 1 | 1 | 0 |
| **General disorders and administration site conditions** | 5 | 2.3 | 2 | 2 | 1 |
| Chills | 1 | 0.5 | 0 | 0 | 1 |
| Device occlusion | 1 | 0.5 | 0 | 1 | 0 |
| Feeling hot | 1 | 0.5 | 1 | 0 | 0 |
| Infusion-related reaction | 1 | 0.5 | 0 | 0 | 1 |
| Pain | 1 | 0.5 | 1 | 0 | 0 |
| Pyrexia | 2 | 0.9 | 1 | 1 | 0 |
| **Hepatobiliary disorders** | 7 | 3.2 | 1 | 4 | 2 |
| Cholestasis | 2 | 0.9 | 0 | 0 | 2 |
| Cytolytic hepatitis | 2 | 0.9 | 0 | 2 | 0 |
| Hepatotoxicity | 1 | 0.5 | 0 | 1 | 0 |
| Hyperbilirubinaemia | 2 | 0.9 | 1 | 1 | 0 |
| **Immune system disorders** | 1 | 0.5 | 1 | 0 | 0 |
| Drug hypersensitivity | 1 | 0.5 | 1 | 0 | 0 |
| **Investigations** | 8 | 3.7 | 3 | 4 | 1 |
| Alanine aminotransferase increased | 2 | 0.9 | 2 | 0 | 0 |
| Aspartate aminotransferase increased | 3 | 1.4 | 3 | 0 | 0 |
| Blood alkaline phosphatase increased | 3 | 1.4 | 0 | 3 | 0 |
| Blood amylase increased | 1 | 0.5 | 1 | 0 | 0 |
| Blood creatinine increased | 1 | 0.5 | 1 | 0 | 0 |
| Blood lactate dehydrogenase increased | 1 | 0.5 | 1 | 0 | 0 |
| Body temperature increased | 1 | 0.5 | 0 | 0 | 1 |
| Gamma-glutamyltransferase increased | 1 | 0.5 | 0 | 1 | 0 |

| | | | | | |
|---|---|---|---|---|---|
| Haemoglobin decreased | 1 | 0.5 | 0 | 1 | 0 |
| Hepatic enzyme increased | 1 | 0.5 | 1 | 0 | 0 |
| Pancreatic enzymes increased | 1 | 0.5 | 1 | 0 | 0 |
| Transaminases increased | 1 | 0.5 | 0 | 1 | 0 |
| **Metabolism and nutrition disorders** | 5 | 2.3 | 4 | 0 | 1 |
| Hyperglycaemia | 2 | 0.9 | 2 | 0 | 0 |
| Hypernatraemia | 1 | 0.5 | 0 | 0 | 1 |
| Hypermagnaesemia | 1 | 0.5 | 1 | 0 | 0 |
| Hyposideraemia | 1 | 0.5 | 1 | 0 | 0 |
| **Nervous system disorders** | 4 | 1.9 | 1 | 3 | 0 |
| Convulsion | 2 | 0.9 | 0 | 2 | 0 |
| Headache | 1 | 0.5 | 1 | 0 | 0 |
| Sensory loss | 1 | 0.5 | 0 | 1 | 0 |
| **Psychiatric disorders** | 3 | 1.4 | 0 | 3 | 0 |
| Agitation | 1 | 0.5 | 0 | 1 | 0 |
| Confusional state | 1 | 0.5 | 0 | 1 | 0 |
| Hallucination, visual | 1 | 0.5 | 0 | 1 | 0 |
| **Respiratory, thoracic, and mediastinal disorders** | 2 | 0.9 | 1 | 1 | 0 |
| Bronchospasm | 1 | 0.5 | 0 | 1 | 0 |
| Dyspnoea | 1 | 0.5 | 1 | 0 | 0 |
| **Skin and subcutaneous tissue disorders** | 6 | 2.8 | 3 | 2 | 1 |
| Alopecia | 1 | 0.5 | 0 | 0 | 1 |
| Erythema | 4 | 1.9 | 2 | 2 | 0 |
| Hyperhidrosis | 2 | 0.9 | 2 | 0 | 0 |
| **Vascular disorders** | 5 | 2.3 | 1 | 3 | 1 |
| Hypertension | 2 | 0.9 | 1 | 1 | 0 |
| Hypotension | 3 | 1.4 | 0 | 2 | 1 |
| **TOTAL** | **80** | | **41** | **29** | **10** |

**Incidence of treatment-related adverse events among patients in the safety population who received anidulafungin only (n = 151)**

| Adverse event | *n* | (%) | Severity | | |
|---|---|---|---|---|---|
| | | | Mild | Moderate | Severe |
| **Blood and lymphatic system disorders** | 4 | 2.6 | 1 | 2 | 1 |
| Anaemia | 1 | 0.7 | 1 | 0 | 0 |
| Eosinophilia | 1 | 0.7 | 1 | 0 | 0 |
| Thrombocytopaenia | 2 | 1.3 | 0 | 2 | 0 |
| Thrombocytosis | 1 | 0.7 | 0 | 0 | 1 |
| **Cardiac disorders** | 4 | 2.6 | 3 | 0 | 1 |
| Atrial fibrillation | 1 | 0.7 | 1 | 0 | 0 |
| Cardiac failure congestive | 1 | 0.7 | 1 | 0 | 0 |
| Myocardial ischaemia | 1 | 0.7 | 1 | 0 | 0 |
| Supraventricular tachycardia | 1 | 0.7 | 1 | 0 | 0 |
| Tachycardia | 2 | 1.3 | 1 | 0 | 1 |
| **Gastrointestinal disorders** | 5 | 3.3 | 3 | 2 | 0 |
| Abdominal pain | 2 | 1.3 | 2 | 0 | 0 |
| Diarrhoea | 3 | 2.0 | 2 | 1 | 0 |
| Vomiting | 2 | 1.3 | 1 | 1 | 0 |
| **General disorders and administration site conditions** | 4 | 2.6 | 1 | 2 | 1 |
| Chills | 1 | 0.7 | 0 | 0 | 1 |
| Device occlusion | 1 | 0.7 | 0 | 1 | 0 |
| Infusion-related reaction | 1 | 0.7 | 0 | 0 | 1 |
| Pain | 1 | 0.7 | 1 | 0 | 0 |
| Pyrexia | 2 | 1.3 | 1 | 1 | 0 |
| **Hepatobiliary disorders** | 3 | 2.0 | 1 | 2 | 0 |
| Cytolytic hepatitis | 1 | 0.7 | 0 | 1 | 0 |
| Hyperbilirubinaemia | 2 | 1.3 | 1 | 1 | 0 |
| **Immune system disorders** | 1 | 0.7 | 1 | 0 | 0 |
| Drug hypersensitivity | 1 | 0.7 | 1 | 0 | 0 |
| **Investigations** | 3 | 2.0 | 1 | 1 | 1 |
| Alanine aminotransferase increased | 1 | 0.7 | 1 | 0 | 0 |
| Aspartate aminotransferase increased | 1 | 0.7 | 1 | 0 | 0 |
| Blood alkaline phosphatase increased | 1 | 0.7 | 0 | 1 | 0 |
| Blood amylase increased | 1 | 0.7 | 1 | 0 | 0 |
| Blood lactate dehydrogenase increased | 1 | 0.7 | 1 | 0 | 0 |
| Body temperature increased | 1 | 0.7 | 0 | 0 | 1 |
| Haemoglobin decreased | 1 | 0.7 | 0 | 1 | 0 |
| **Metabolism and nutrition disorders** | 3 | 2.0 | 2 | 0 | 1 |
| Hyperglycaemia | 1 | 0.7 | 1 | 0 | 0 |
| Hypernatraemia | 1 | 0.7 | 0 | 0 | 1 |
| Hyposideraemia | 1 | 0.7 | 1 | 0 | 0 |

| | | | | | |
|---|---|---|---|---|---|
| **Nervous system disorders** | 3 | 2.0 | 1 | 2 | 0 |
| Convulsion | 2 | 1.3 | 0 | 2 | 0 |
| Headache | 1 | 0.7 | 1 | 0 | 0 |
| **Psychiatric disorders** | 2 | 1.3 | 0 | 2 | 0 |
| Agitation | 1 | 0.7 | 0 | 1 | 0 |
| Hallucination, visual | 1 | 0.7 | 0 | 1 | 0 |
| **Respiratory, thoracic, and mediastinal disorders** | 2 | 1.3 | 1 | 1 | 0 |
| Bronchospasm | 1 | 0.7 | 0 | 1 | 0 |
| Dyspnoea | 1 | 0.7 | 1 | 0 | 0 |
| **Skin and subcutaneous tissue disorders** | 3 | 2.0 | 0 | 2 | 1 |
| Alopecia | 1 | 0.7 | 0 | 0 | 1 |
| Erythema | 2 | 1.3 | 0 | 2 | 0 |
| **Vascular disorders** | 4 | 2.6 | 1 | 2 | 1 |
| Hypertension | 2 | 1.3 | 1 | 1 | 0 |
| Hypotension | 2 | 1.3 | 0 | 1 | 1 |
| **TOTAL** | **52** | | **25** | **19** | **8** |

**Figure S2. Kaplan–Meier estimate of survival to day 90 in modified intent-to-treat patients**

Day 1 represents the first day of anidulafungin therapy.

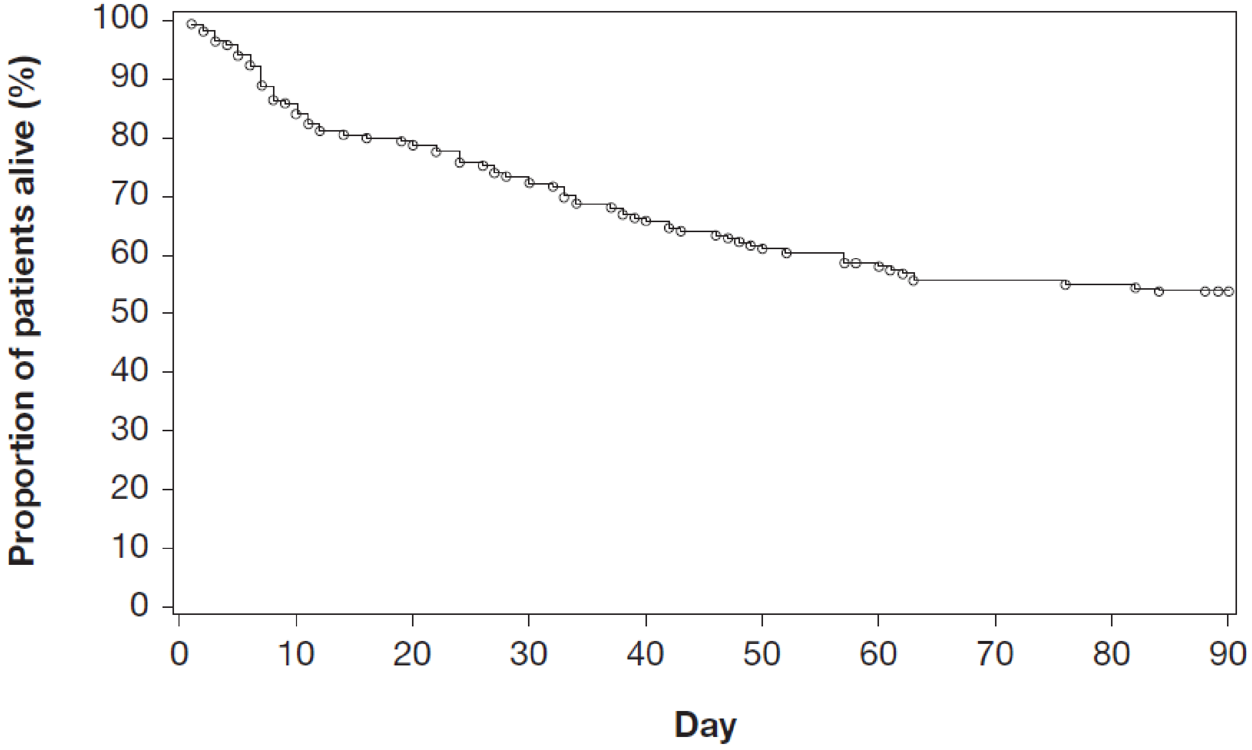

Supplement: Supplementary file 1 [file clm0018-0680-SD1.pdf]
